# The Role of Extracellular Vesicles in the Development of a Cancer Stem Cell Microenvironment Niche and Potential Therapeutic Targets: A Systematic Review

**DOI:** 10.3390/cancers13102435

**Published:** 2021-05-18

**Authors:** Thomas J. Brown, Victoria James

**Affiliations:** Biodiscovery Institute, School of Veterinary Medicine and Science, University of Nottingham, Loughborough LE12 5RD, UK; stxtb10@exmail.nottingham.ac.uk

**Keywords:** extracellular vesicle, exosome, cancer, cancer stem cell, microenvironment, stem cell, therapeutics

## Abstract

**Simple Summary:**

Cancer stem cells (CSCs) are cancer cells that possess traits usually attributed to stem cells. An increase in CSCs can lead to more rapid cancer progression, treatment resistance and the increased likelihood of recurrence. To promote CSC survival and associated cancer progression, cancer cells enter into reciprocal crosstalk with the surrounding tissue environment, as well as with distant metastatic sites. This mechanism of communication relies, in part, on secreted factors, of which extracellular vesicles (EVs) are thought to have a critical role. This systematic review evaluates the current knowledge of cancer communication via EVs to alter the microenvironment to increase the survival and maintenance of CSCs. A total of 16 studies spanning the EV content, pathway alterations and CSC-targeting treatments provide new insights into how EVs mediate CSC traits and identify the gaps in our understanding of how modulation of the microenvironment plays a key role.

**Abstract:**

Cancer stem cells (CSCs) have increasingly been shown to be a crucial element of heterogenous tumors. Although a relatively small component of the population, they increase the resistance to treatment and the likelihood of recurrence. In recent years, it has been shown, across multiple cancer types (e.g., colorectal, breast and prostate), that reciprocal communication between cancer and the microenvironment exists, which is, in part, facilitated by extracellular vesicles (EVs). However, the mechanisms of this method of communication and its influence on CSC populations is less well-understood. Therefore, the aim of this systematic review is to determine the evidence that supports the role of EVs in the manipulation of the tumor microenvironment to promote the survival of CSCs. Embase and PubMed were used to identify all studies on the topic, which were screened using PRISMA guidelines, resulting in the inclusion of 16 studies. These 16 studies reported on the EV content, pathways altered by EVs and therapeutic targeting of CSC through EV-mediated changes to the microenvironment. In conclusion, these studies demonstrated the role of EV-facilitated communication in maintaining CSCs via manipulation of the tumor microenvironment, demonstrating the potential of creating therapeutics to target CSCs. However, further works are needed to fully understand the targetable mechanisms upon which future therapeutics can be based.

## 1. Introduction

Cancer is one of the leading causes of death worldwide, second to only cardiovascular disease. Cancer cases are predicted to continue to rise, from 18.1 million new cases and 9.5 million cancer-related deaths worldwide in 2018 to 29.5 million new cases per year and the number of cancer-related deaths to 16.4 million by 2040 [1,2]. There is a critical need to better understand the mechanisms of cancer progression to develop more effective management strategies.

### 1.1. Cancer Stem Cells

**Figure 1:**
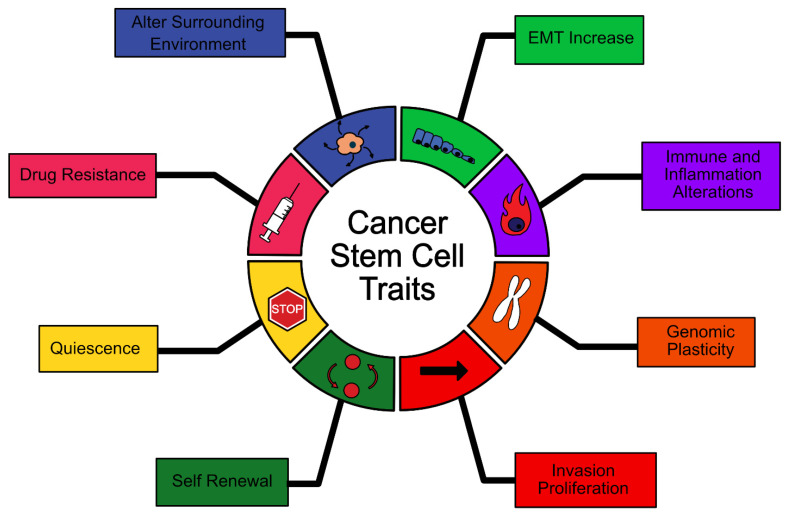
Summary of cancer stem cell traits.

Many of the same abilities that define stem cells can be observed to be shared by cancer stem cells (CSCs), manifesting in the capacity for unlimited self-renewal, the ability to enter quiescence, increased drug resistance, increased EMT, alteration of the immune response, alteration of the surrounding microenvironment, increased cell plasticity and increased proliferation/invasion (Figure 1). These CSC properties lead to an increased capacity for treatment resistance, immune system evasion/manipulation, invasion, metastasis, progression and recurrence [3,4,5,6,7].

Much like stem cells, CSCs depend on their microenvironment niche to assist in their development and maintenance. For example, brain tumors can induce a brain tumor-initiating cell phenotype within a hypoxic niche to increase the resistance to hypoxic and acidic stress [8], whereas the stem cells residing in the avascular niche show increased the cytokine release, resulting in inflammation. This can even vary within the microenvironment niche itself. For example, within the bone, CSCs on the periphery of the bone marrow niche can be mobilized to enter into the circulation, whilst CSCs within the central bone marrow regions may be induced into quiescence. This is particularly relevant in cancer metastasis, whereby, once residing in the metastatic microenvironment, CSCs may need to exhibit different epigenetic and transcriptional signatures for their maintenance, modification of the niche and progression to metastases.

CSCs communicate with surrounding cells by a variety of signaling mechanisms, allowing the tumor to have reciprocal communication within itself and the surrounding microenvironment. This communication allows for the manipulation of the surrounding cells dependent on the current requirements of the cancer. Crosstalk between the surrounding cells and the cancer can be between many different cell types, altering their activity, phenotype and their presence in the CSC niche [9,10,11,12]—for example, the conversion of fibroblasts into cancer-associated fibroblasts (CAFs) or the creation of an immune privileged area.

Despite our existing knowledge of the role of CSCs in cancer initiation and progression, the full repertoire of the signaling mechanisms used to maintain the CSCs and mediate these effects remains to be elucidated. More recently, extracellular vesicles (EVs) were identified as an important part of the mechanism of communication between the cancer and its microenvironment.

### 1.2. Extracellular Vesicles

Extracellular vesicles are a class of small membrane-bound organelles with various functions, such as cell-to-cell communication or as a cellular waste route. EVs are not one homogenous group; the term encompasses at least three major subsets of vesicle: exosomes, microvesicles (MVs) and apoptotic bodies. These groupings are determined by either the physical characteristics of the vesicles (e.g., size) or the process of their biogenesis [11].

#### 1.2.1. Exosomes

Exosomes are the most researched class of EVs, as they are thought to facilitate cell-to-cell communication. At 30–150 nm, they are the smallest class of EV and have an endosomal origin. They are formed from the budding of multivesicular bodies to form intraluminal vesicles (ILV), which are then released as exosomes into the extracellular space via the fusing of ILVs with the plasma membrane. Biogenesis is achieved either through ESCRT-dependent or -independent pathways. The exosome content often reflects that of the parental cell, containing RNA, DNA, lipids and proteins [12]. Interestingly some components in the exosome cargo can be enriched to many times the concentration found in the parental cell, suggesting selective exosome cargo loading, a mechanism further supported by the presence of proteins associated with cellular sorting processes [13]. This potentially selected exosome cargo has also been shown to be biologically active in the recipient cells [14].

#### 1.2.2. Microvesicles

Microvesicles (also termed shedding vesicles, ectosomes, shedding bodies and microparticles), instead of an endosomal origin, originate via direct budding from the plasma membrane, which, when excised, is released into the surrounding extracellular space. Typically, microvesicles are thought to span 100 nm to 2000 nm, although, when a cell becomes cancerous, it can release “oncosomes”, which are classified as MVs yet are much larger, up to 10,000 nm, due to biogenesis dysregulation [15]. Much like exosomes microvesicles can contain RNA, DNA, lipids and proteins, as well as whole or parts of cellular organelles [16]. Given the overlap in size, the separation of exosomes and microvesicles is technically challenging. Therefore, exosomes and microvesicles are often studied as one population under the collective term of EVs or small vesicles.

#### 1.2.3. Apoptotic Bodies

Apoptotic bodies are created exclusively when a cell undergoes programmed cell death (apoptosis) and begins to physically break down. As a cell undergoes apoptosis, it releases apoptotic bodies that are filled with the broken-down contents of the cell. The plasma membrane directly buds and excises around the contents and immediate surrounding cytoplasmic fluid, forming an apoptotic body from 500 nm to 5000 nm, allowing apoptotic bodies to act as a waste route and packaging the internal contents of the cell for removal. This process means that the apoptotic bodies can be filled with all components of the cell, including DNA, RNA, lipids, proteins and organelles [17], aiding the apoptotic clearance and preventing autoimmune diseases, although recent studies are beginning to explore a possible role for apoptotic bodies in cell-to-cell communication.

#### 1.2.4. Other EVs

Extracellular vesicles are not only are found within animal cells; they are an inherent feature of all eukaryotes, bacteria and archaea [18]. Interestingly, in bacteria, there is evidence of the existence of exosomes in the size range of 30–150 nm. However, bacteria lack necessary endosomal organelles to create exosomes, yet are still able to make vesicles within the size range, suggesting potential subpopulations of exosomes within the 30–150-nm size range.

Prostasomes are extracellular vesicles of 50 nm–400 nm that are released into the seminal fluid by the prostate gland [19]. Similar to other EVs, the membranes of prostasomes consist of a phospholipid bilayer. However, the prostasomes surface is enriched with lipids in an organized structure. The main roles of prostasomes are in the survival of sperm cells in reproduction, either in increasing motility or in protection from the female’s immune system [20].

### 1.3. Extracellular Vesicles in Cancer

The communication between the cancer cells and their surrounding environment has been found to be important for cancer progression. Previously, this was thought to be achieved via direct cell–cell contact or via soluble intermediaries, until the role of EVs in cell-to-cell communication emerged. EVs can bind to recipient target cells, resulting in either a ligand–receptor-mediated response in the target cell or the uptake of the EV into the recipient cell for processing [19]. EVs entering the recipient cells have the potential to release biologically active cargos of RNA, DNA, proteins and organelles to trigger a response in the recipient cell [21,22]. Cancer cell EVs can mediate crosstalk between the cancer cells and other surrounding nonmalignant cell types, acting alongside direct cell-to-cell interactions and secreted soluble factors. When exposed to EVs derived from aggressive cancer cells, the recipient cancer cells gain increased metastatic/invasive potential and increased the stemness properties or, in the case of nonmalignant stromal cells, an altered phenotype to become supportive of cancer progression.

As EVs are stable in most extracellular fluids and are subject to minimal degradation when in the circulation, they provide an effective means of communication between the tumor and cells located in distant sites of the body. This ability enables cancer cells to begin a process of early crosstalk with potential metastatic sites, primarily creating a microenvironment favorable for later metastasizing cancer cells. The distal communication between cancer and its metastatic sites via EVs may elucidate some aspects of organotropism found in metastasizing cancers. For example, osteoblasts and brain cells are targeted by EVs derived from prostate and breast cancers, respectively, causing the creation of a premetastatic niche [23]. Studies such as Hoshino et al. 2015 hypothesized a link between organotropism and the EV cargo, such as integrins [24]. However, the mechanisms that dictate EV targeting are still largely unknown. This same ability of stable survival within the extracellular space, alongside protection of the internal cargo, makes them promising for future biomarkers [25].

EVs can target a multitude of cell types, resulting in different responses to the messages being communicated. For example, endothelial cells targeted by cancer EVs are reported to increase their vascular permeability, thereby assisting in cancer metastasis and progression [26], whilst fibroblasts can be transformed by exposure to cancer EVs into cancer-associated fibroblasts with protumorigenic and proangiogenic effects [27]. EV communication between cancer cells and the immune system can have both pro- and antitumor effects. Where antitumor effects occur, there is often a disruption of the immune activity and inflammatory processes, including the impairment of CD8+ lymphocyte activation and recruitment of regulatory T cells [28,29], promoting a premetastatic niche formation via immunosuppression [7]. Immune cells, especially those adapted by the tumor microenvironment, can also release EVs that have a protumor effect [30]. These “extracellular vesicle-transformed” noncancer cells, of both the immune and stromal compartments, can actively modify the cancer microenvironment, increasing tumor progression and metastasis.

CSCs have a key role in the progression of cancer, acting to increase the growth, metastasis, chemoresistance and potential future relapse. As CSCs are dependent on the microenvironment, which can be modulated by EVs, the mechanisms employed by EVs may provide important insights into therapeutically targetable mechanisms to clear the CSCs at tumor sites. This systematic review evaluates the research on EVs in the progression and alteration of the tumor microenvironment, highlighting the existing findings and gaps in our knowledge.

## 2. Results

**Figure 2:**
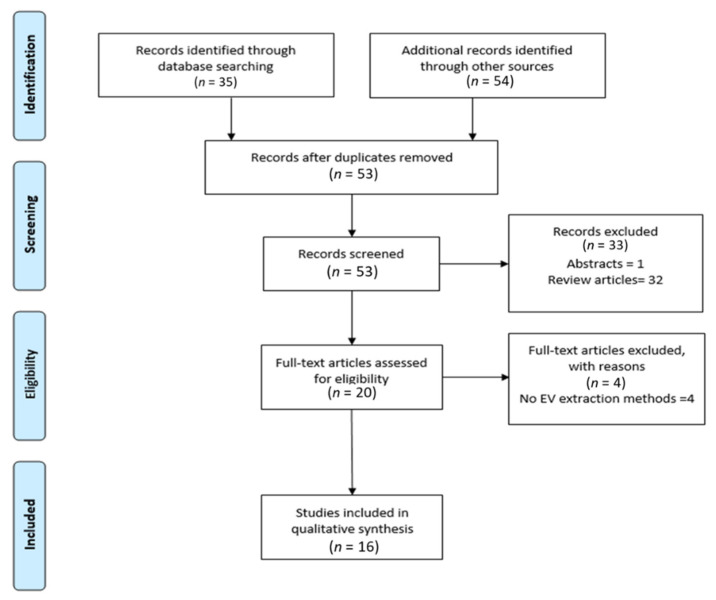
Flow chart demonstrating the application of the Preferred Reporting Items for Systematic Reviews and Meta-Analyses (PRISMA).

The use of our search terms in EMBASE and PubMed identified 54 and 35 publications, respectively. When duplicates were removed (*n* = 26), this left 53 unique publications. These 53 studies were screened for eligibility via title and abstract, and 33 were removed, leaving 20 articles that were evaluated in full. This resulted in the further removal of four studies, as there was no inclusion of the EV isolation methods, leaving 16 eligible studies to be included in the review. A detailed diagram of the review process is represented in Figure 2.

**Figure 3:**
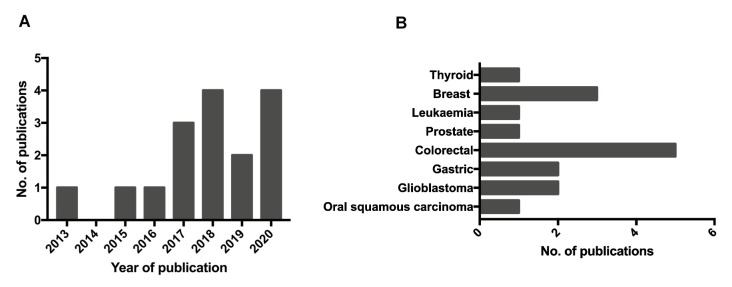
Distribution of eligible publications by year and cancer type. (**A**) Eligible studies were published between 2013 and 2020, with the largest proportions contributed between 2017 and 2020. (**B**) The representation of cancer types per eligible study, demonstrating a prevalence of studies in the colorectal and breast cancer fields.

The publications span from 2013 to 2020, with only one study meeting the eligibility criteria in 2013, rising to four studies in 2020 (January–July), as seen in Figure 3A. Their papers were spilt between cancer types, with a concentration around colorectal cancer, as seen in Figure 3B.

The studies were divided into three categories: extracellular vesicle cargo, activated pathways and CSC-targeted therapeutics. Four studies focused on extracellular vesicle cargos, eight studies focused on activated pathways and the final four studies focused on CSC-targeted therapeutics. The studies in each group were assessed on quality, and the summaries can be found in Supplementary Tables S1–S3.

### 2.1. Extracellular Vesicle Cargo

In total, there were four original research studies that investigated the internal contents of EVs and their links to the increasing stemness in cancer [31,32,33,34]. All four studies initially confirmed that treatment with EVs from increasingly aggressive metastatic cell lines increased the cancer cell stemness and EMT status, as assessed by typical EMT and stemness markers.

Two of the four studies used omics-level approaches [31,33] to identify the transcript levels and protein contents [33]. These studies reported a range of both miRNAs or proteins enriched within EVs isolated from cells showing greater stem cell attributes or metastatic potential. Ramteke et al. 2015 identified a range of 160 proteins, including β catenin enriched within EVs isolated from prostate cancer, whilst Donnarumma et al. 2017 found only three miRNA significantly enriched breast cancer cell lines (miR-21-5p, miR-378e and miR-143-3p) [31,33].

The two remaining studies used a more targeted qPCR approach to determine the levels of long noncoding RNAs (lncRNAs) and miRNAs. Razmkhah et al. 2017 and Hardin et al. 2018, from EVs extracted from acute myeloid leukemia and anaplastic thyroid carcinoma, respectively, demonstrated an enrichment of targets miR-21 and lncRNA linc-ROR in EVs of cell lines showing an increased metastatic potential and stemness. Both studies also directly analyzed the effects of the EVs on cancer-associated fibroblasts to determine the potential effects on the microenvironment [31,33], with both papers reporting a reciprocal signaling mechanism from CSCs to increase CAF transformation [31,33] and form CAFs to enhance CSC properties [31].

The quality score for the studies determining the effect of EV cargo on the microenvironment and their effect on CSC ranged from 0.46 to 0.77 (full breakdown in Table 1). Hardin et al. 2018 scored highly due to rigorous EV isolation and the evaluation methods gaining within eight out of eight of these categories. Conversely, despite an in vivo focus, Donnurumma et al. 2017 had no noted EV characterization methods (isolation methods were included), which lowered the overall score (Supplementary Table S1).

### 2.2. Activated Pathways

**Figure 4:**
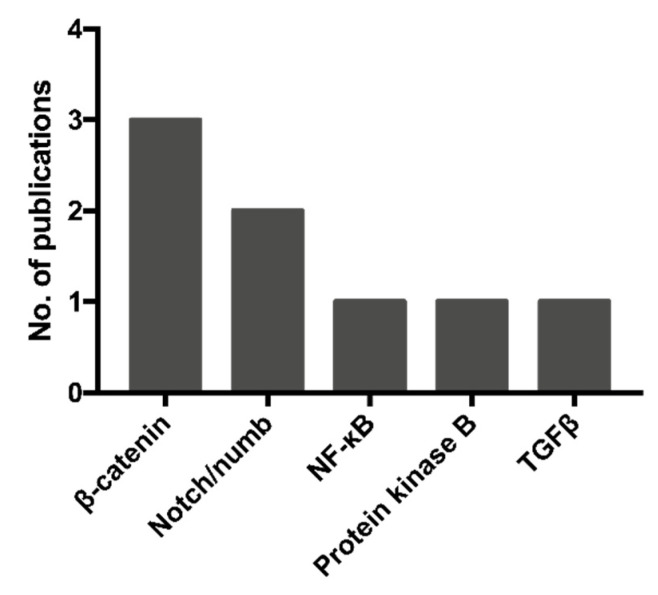
A summary of the pathways modified by CSC-EVs, as reported in the 8 evaluated studies (31–38). The Wnt/β-catenin and Notch pathways featured in the majority (5/8) of the studies reviewed.

Eight articles studied the alterations in the activation of the cellular pathways due to EV exposure, identifying five key regulatory pathway targets (summarized in Figure 4) [35,36,37,38,39,40,41,42].

Three studies focused on a single pathway, Wnt/β-catenin [35,40,42]. Pathway activation was identified by quantification of the protein β-catenin [35,41] or one of its subsequent downstream elements, such as CD44, CyclinD1, CyclinD3 and C-myc [40]. All three studies showed that at least one component of the β catenin pathway was altered, with Mao et al. 2017 demonstrating the changes in additional the protein components when the cancer cell stemness was increased [40]. Two further studies focused on the notch1/numb signaling pathway, a β-catenin-related pathway, showing that the inhibition of NOTCH via NUMB increased the cancer stem cellness [38,42]. The remaining three studies reported EV modulation of the TGF-β [39], NF-kB [37] and protein kinase B [36] pathways, confirming the increased activation of each pathway and an increase in the markers related to stemness.

Interestingly, the upregulation of TGF-β, NF-kB and protein kinase B by EVs was also connected to altered immune cell function, confirming that a key element in creating a niche favorable to CSC survival is in the alteration of the immune response. This was explored further by Hwang et al. 2019 and Cheng et al. 2019, who determined the effect of pathway alterations upon neutrophils from colorectal cancers [35,37]. Hwang elucidated a multistep process of crosstalk between the stem cells and neutrophils, whereas Cheng found an increase in tumor-filtrating CD66(+) neutrophils, causing a decreased number of tumor-infiltrating CD8(+) T cells. Both confirmed that exosomal RNAs have a role in manipulating the immune system, providing an immunosuppressive tumor microenvironment.

Two of the eight studies also looked at the effects of EVs on pathway activation in cancer-associated fibroblasts (CAFs), demonstrating that CSC-to-CAF communication acts as a route to further increase the stemness. The specific role of fibroblast EVs in CSC-to-CAF communication in colorectal cancer was further confirmed by Liu et al. 2020, demonstrating EV-mediated reciprocal crosstalk between CSCs and the microenvironment [39].

The quality score between the studies determining the pathway activation by CSC EVs to increase the stemness properties were reasonably consistent, ranging from 0.46 to 0.69, with an average of 0.61. Universal scores for EV isolation were low at 1 due to the use of ultracentrifugation alone; however, this was compensated by all the articles that gained 3 or 4 for the EV evaluation, with the exception of Gu et al. 2016 [36]. A full breakdown can be found in Supplementary Table S2.

### 2.3. CSC Targeted Therapeutics

The effect of treatments on cancer cell stemness mediated by EVs was investigated in four studies [43,44,45,46]. Three of the four studies began by confirming that the EVs increased the cancer cell stemness via tumor sphere formation, enhanced metastatic capabilities or using stemness markers.

Ovatodiolode (OV), a bioactive component of *Anisomeles indica*, was tested for its ability to suppress oral squamous cell carcinoma (OSCC) progression [43]. OV was found to reduce the miR21-5p, STAT3 and mTOR levels in EVs produced by OSCC CSCs, resulting in re-sensitization of the OSCC cells to cisplatin and the inhibition of the transformation of the surrounding fibroblasts. Similar is OV Pacritinib, an FDA-approved chemotherapeutic, was also found to lower the levels of miR21 found in the EVs produced by glioblastoma cells. This resulted in an increase in PDCD4 expression within the glioblastoma cells and subsequent increased sensitivity to Temozolomide [44].

An additional FDA-approved drug, the chemotherapeutic Fludarabine, was identified as inhibiting the cross-communication mediated by EVs between CSCs and the local microenvironment on breast cancer. Xing et al. 2018 demonstrated that breast cancer cells expressing low levels of the lncRNA Xist produced EVs enriched in the expression of miR-502. These EVs triggered the polarization of microglia that resulted in the suppression of T-cell proliferation through the upregulation of immunosuppressive cytokines [46]. Fludarabine, was found to be lethal to breast cancer cells expressing low levels of Xist, blocking this EV-mediated mechanism of communication and the subsequent immune suppression, resulting in a reduction in brain cells [46].

The targeting of EV cargos in the stroma rather than CSCs was also investigated by Gernapudi et al. 2015., who determined that the natural compound Shikonin drove pre-adipocytes to release EVs containing high levels of miR-140. When applied to ductal carcinoma in situ (DISC) cells, miR-140-enriched EVs reduced the Sox9 expression in the DISC cells and blocked cancer progression [43].

Quality scores for the studies assessing possible treatments to block the EV-mediated effects on cancer cell stemness and cancer progression ranged from 0.38 to 0.69. Studies evaluating the FDA-approved drugs scored highly in each assessment criteria due to large sample sizes and the use of the drug, allowing for human samples and databases to be included, whilst the lowering scorings studies lacked extensive EV isolation and evaluation methods. A full breakdown can be found in Supplementary Table S3.

## 3. Discussion

### 3.1. Extracellular Vesicle Cargo

A large proportion of the cell-to-cell communication mediated by the vesicles is believed to be via the cargo. The EV cargos contain different proteins, lipids, RNAs and DNAs, which may be representative of the parental cell or show a significant enrichment of certain molecules. The enrichment of some proteins and RNAs within EVs has been shown to cause modulation of the microenvironment and premetastatic niche formation, via increasing the stemness and alteration of surrounding the stromal and immune cells. The contents of the EVs have become of increasing interest, as others have shown DNA [47], RNAs [48] and proteins [21] to have a biologically active role after delivery to recipient cells from EVs.

Four studies determined the contents of the EVs and their effects on cancer stem cells, finding that either specific RNAs or proteins were enriched in the cargos of EVs produced by metastatic cancer cells compared to their less malignant counterparts and affected the CSCs and tumor progression [31,32,33,34]. Interestingly, despite the different approaches, two independent studies found miR-21 to be one of the highest increased cargo components of cancer cell EVs [31,34]. Although no potential targets or mechanisms of action were determined in these studies, the targets of miR-21 in tumorigenesis have been well-dissected within the literature.

miR-21 has been shown to be involved in many pathways, including acting as a prosurvival factor within antiapoptotic pathways [49,50] across multiple cell types. Specific targets of miR-21 include the matrix metalloproteinases (MMPs) and intracellular Toll-like receptors, such as TLR7 or TLR8 [51], suggesting miR-21 involvement within the extracellular structures and innate immunity. Together, with the evidence from the EVs, the data suggests that vesicular miR-21 can alter the stemness via the modulation of the surrounding microenvironment through both matrix and immune cell manipulation.

Ramteke et al. analyzed the protein contents of the EVs from prostate cancer and found that most of the proteins were involved with either cytoskeleton signaling or in cell-to-cell junction signaling. This further supports the hypothesis that the EV content has a key function in creating and maintaining a microenvironmental niche for CSCs. Additionally, this highlights that, much like in a complex disease model, there may be many elements of the EV cargo, including proteins and RNA, which cumulatively affect the stemness properties of the cancer cells and their surrounding microenvironment.

The altered contents of cancer EVs, particularly of some highly enriched components such as miR-21, has led to the exploration of the use of EVs as biomarkers. The current diagnostic and prognostic markers for cancer lack either specificity or sensitivity, limiting their utility. For example, within prostate cancer, the prostate-specific antigen (PSA) is used as a cancer marker but has a high false-positive rate for diagnosis [52]. EVs have the potential to replace the current cancer markers and are easily accessible in a range of biofluids. Although no EV-based biomarkers are currently used for diagnostic or prognostic purposes, there are multiple studies assessing the clinical use of EVs in cancer, including a clinical trial of Gylpican-1^+^ (GPC1^+^) EVs for the diagnosis of pancreatic cancer [53]. The topic of the clinical use of EVs as biomarkers has been comprehensively reviewed by Zhou et al. 2020 [54]. Continued research in this area is likely to yield a range of both EV-based biomarkers to provide diagnostic and prognostic information.

Taken together, these studies demonstrated that cell-to-cell communication and manipulation of the microenvironment via stemness is likely, at least in part, to be dependent upon the cargo of the EVs. Further studying of the functional effects of the EV cargo on recipient cells may provide the key to understanding the mechanisms mediated by EVs to affect cancer stem cells.

### 3.2. Processes Activated by Cancer EVs

The enriched cargo elements of EVs facilitate their ability to communicate between cells, resulting in increased cancer cell stemness. As the EV cargo is generally biologically active when released, the functional studies of EV recipient cells showed a range of altered pathway signaling and altered gene expression. Eight studies investigated the potential pathways activated by cancer cell EVs and confirmed an increased stemness that was directly due to EV exposure.

Three studies focused on a single pathway, Wnt/β-catenin [35,40,42]. All three papers noted an increased activation of the β catenin pathway, directly causing an increase in cancer cell stemness. Due to the β-catenin involvement in embryonic development, there are multiple processes that are thought to contribute to the β-catenins effects, including mediating cell fate specification, cell cycle progression and cell migration [55]. As well as modulating the infiltration of other cell types into the tumor microenvironment, such as T cells [56]. Although β-catenin acts via a range of downstream elements, it is the activation by transcriptional repressors, such as snail1 and snail2, that appears key to mediating the CSC properties [56].

Additionally, all three studies linked increased the activation of the target pathway to a specific biologically active component of the EV cargo. These were miRNAs (miR-146a-5p and miR-142-3p) [35]; lncRNAs (H19) [41] and proteins (ubr2) [40], with each showing pathway activation directly correlating with the biological active component. Interestingly, Ren et al. 2018 found that lncRNA H19 acted as a sponge specifically for miR-141, leading to increased activation and stemness [41], suggesting that certain miRNAs are vital in increasing cancer cell stemness via the β-catenin pathway [31,36], alongside the other cargo components.

In addition to the studies included within this systematic review, other investigations have found that β-catenin and its downstream elements play a vital role in cancer stem cell survival in multiple cancer types [57,58] and have a potential function in adapting metastatic sites such as the bone marrow [59].

Two further studies reported activation of the Notch/Numb pathway. Whilst not indicated in either study, the Notch/Numb signaling pathway is directly linked to β-catenin. As the notch1 expression is dependent on the β-catenin pathway activation [60], resulting in five out of the eight papers focused on the codependent pathways of Notch1/Numb and β-catenin [35,38,40,41,42]. These findings combined strongly suggest EV-mediated β-catenin as a critical factor in increasing the CSCs and cancer survival.

Whilst not all of the studies directly investigated the potential of a specific pathway and activating the component within the EVs cargos, four studies did investigate if exposure to cancer EVs in general was correlated with the target pathway activation [35,38,40,41]. Even though the pathways were confirmed to be activated by EV exposure, further studies should be conducted to identify if the EV cargo is responsible for the pathway activation or if other elements of the process or if EV exposure is responsible for the changes observed, such as an altered response due to physical contact with the EV or changes in the local extracellular matrix.

Of note, two studies also investigated the communication from CAFs to CSCs via the EV cargo elements [39,41]. Each study showed an increase of either TGF-β or the β-catenin pathway; within both, this resulted in an increase of CSC properties with Ren et al., also showing a subsequent increase in resistance to radiotherapy. This data indicates the presence of reciprocal communication between the surrounding cells in the microenvironment and the cancer. Which is supported by Sansone et al. 2017 finding the role of content of CAF-derived microvesicles activating the Notch pathways via miR-221 [61]. Although this was only shown through CAFs, it illustrates the role played by the tumor microenvironment to maintain and increase the properties of CSCs.

### 3.3. Treatment

CSCs can increase the resistance to chemotherapy and radiotherapy. As discussed previously, EVs can increase the stemness of cancer cells to in part mediate the resulting resistance to therapy. Currently cancer therapeutic agents are non-selective for CSCs [62]. However, the recent studies reviewed in our report indicate the potential for CSC directed therapies that target either pathways altered by CSC EVs or by targeting the CSC EV content directly.

Of the four studies reviewed, three confirmed the ability of EVs to increase CSC markers and enhance metastatic capabilities [43,44,45,46], with two studies investigating the effect of EVs on resensitization after the development of chemoresistance. OV treatment of oral squamous carcinoma led to a significant decrease of the loading of key components of the EV cargo that were attributed to the transformation of the surround stromal cells, specifically fibroblasts. These reduced components were primarily those found increased in cancer EVs such as miR-21 and β-Catenin, all of which had been linked with CSC activity previously [35,38,40,41,42] These OV induced EV changes led to resensitization of tumors to cisplatin, thought to be through the restoration of the pre-cisplatin treatment microenvironment.

Similarly, Chuang et al., showed glioblastoma gained temozolomide resistance due to altering the miR-21 content of EV [44]. Delivery of EV miR-21 converted the macrophages from an M1 to an M2 (protumor) phenotype. The use of Pacritinib reversed this change, returning macrophages to the M1 phenotype and, subsequently resensitizing the glioblastoma to temozolomide.

Together these studies suggest that targeting the activity of EVs, has the potential to greatly enhance the effectiveness of chemotherapies, even in tumors previously found to be resistant. Further research is needed to determine if this type of targeting prevents alterations of the microenvironment stops the development of chemoresistance as well as providing a mechanism of resensitization.

The final two studies evaluated Shikonin and Fluarabine, both agents were shown to have anti-breast cancer CSC properties. Shikonin directly targets the communication between breast cancer cells and preapodocytes via interruption of the miR-140/SOX9 signaling that is facilitated through EVs. This signaling mechanism has been previously reported to mediate an immune privileged area in both the immediate surrounding environment and in metastatic niches [63]. Interrupting this communication mechanism reduced the aggressive cancer phenotype and chemoresistance by reduced stemness and preventing alterations within the microenvironment.

Of the compounds tested, it is of note that Fludarabine showed an IC50 value for breast cancer cells ten times lower than the clinical dose used in treating leukemia, for which the drug originally gained FDA approval [46]. Additionally, Fludarabine was effective in treating brain metastasis without any noted side effects in vivo. As the two studies which evaluated approved FDA drugs (Fludarabine and Pacritinib) had access to human databases, both studies were able to identify lower relapse rates, which can potentially be linked back to the treatments targeting CSCs [44,46]. Suggesting other chemotherapies may prove more effective when repurposed against CSCs in different tumor types. In addition, these studies also indicate there may be the potential to create newer combination therapies which can target the cancer microenvironment, CSCs and the primary cancer.

## 4. Materials and Methods

### 4.1. Sources and Searches

This review was conducted under the Preferred Reporting Items for Systematic Reviews and Meta-Analyses (PRISMA) guidelines. Publications were searched for using EMBASE and PubMed and results were up to July 2020 with the search terms (Exosomes OR extracellular vesicles OR microvesicles OR microparticles OR ectosomes) AND (cancer) AND (microenvironment) AND (stemness).

### 4.2. Study Selection

This systematic review was conducted to determine the role of extracellular vesicles in the development of a microenvironment favorable to cancer stem cells. Studies which identified one or more of: EV content, EV effect on recipient cells (pathway activation) or a therapeutic approach to targeting CSC via mechanisms facilitated by EVs. With only publications which contained primary research were included.

Publications were included in this review if they met the criteria summarized Table 2.

### 4.3. Study Quality Assessment

To assess the quality of each of the publications viewing the role of EVs in CSCs, each study was assessed on three key components: EV isolation methods, EV characterization and the samples analyzed. A summary of quality score criteria can be found (Table 1).

The EV isolation methods were graded to merit the quality/purity of the EVs extracted (n/4). As any studies with no data on isolation methods were removed during screening, there was no score of 0. A score of 1 applied to unbuffered ultracentrifugation only, a score of 2 for size exclusion extraction, a score of 3 for any previous isolation by ultracentrifugation or seize exclusion chromatography followed by bead extraction. Finally, as there is no gold standard for EV extraction, if one of the previous methods was used with additional or novel methods to improve purity, a score of 4 was given.

For the characterization of EVs a score of 0 was given if there was no method of characterizing the quality of the EVs collected. A score of 1 was given if nanoparticle tracking or dynamic light scatter was used; a score of 2 was given if nanoparticle tracking was used alongside a western blot or recommended EV markers; a score of 3 was given if particle tracking was used with electron microscopy. Finally, a score of 4 was given if at least two of the other methods were used alongside a novel technique (i.e., FACS).

For assessing the quality of the samples used to analyze the role of EVs in CSCs, a score from 1-5 was used. A score of 1 was given for 2D in vitro models; a score of 2 for 3D in vitro models; a score of 3 for use of in vivo animal models. A score of 4 was awarded for the integration of human patient samples or databases in addition to other models. Finally, a score of 5 was given for the use of both in vivo human samples and databases.

## 5. Conclusions

There is a growing body of research determining the mechanisms that govern the interaction between CSCs and their surrounding environment. The recent entry of EVs into this field has opened new lines of investigation that play a key role in cell-to-cell communication alongside other more traditionally studied signaling processes. This review provided an in-depth coverage of the topic in relation to EVs. It is important to note that the studies for which the main focus is not CSCs did not incorporate by the methodology applied, these include those by the Lyden group and others. However, where these studies pertain to the findings of this analysis, the data support our overall conclusions as indicated in the discussion. Despite recent advances in EV research, there is often still a focus on the miRNA content of EVs and the use of targeted approaches that study only a single EV cargo molecule in isolation. Future studies in this area could focus on the other RNAs that are present and often more predominant in cancer EVs through the use of global transcriptome methods. As determined within this review, the majority of research on pathway alternations focuses on the β catenin/NOTCH signaling pathway. More in-depth analysis, particularly longitudinal studies that look at the effects of repeated EV exposure over time, may present other pathways specific for CSC maintenance and microenvironment manipulation for further investigation. The field currently views the reciprocal cross talk between CSC and surrounding cell types as an important element of microenvironment modulation and increasing cancer cell stemness. Notably neutrophils and CAFs are two elements demonstrated to provide a key role in EV-modulation of the tumor microenvironment. The inclusion of other critical components such at the ECM or tumor associated macrophages may provide an interesting future direction to expanded upon. An analysis of the spatial efficacy of EVs may also prove to be an important factor when ascertaining their potential to modulate immune and stromal cell function in vivo. Therapeutically targeting CSCs currently shows promise to treat metastasizing cancer and reduce recurrence. This review highlights the potential of repurposing two approved cancer drugs, which may potentially be effective in other cancer types with decreased toxicity due to the direct targeting of CSCs and their EVs. Additionally, bioactive components of plants or herbs pose another potential avenue to target CSCs EVs. In conclusion, the field of CSC interaction with their microenvironment via EV-facilitated communication is a promising field. As research grows, exploring EV content, and their target pathways, in relation to the interplay between CSCs and the tumor microenvironment presents additional targets for improved therapeutic intervention.

## Figures and Tables

**Table 1:** Quality assessment score summary.

Quality Criteria	Score					
	0	1	2	3	4	5
EV isolation methods	-	Ultracentrifuge only	Size exclusion extraction	Bead based extraction	Additional methods (sucrose cushion, FACS, novel methods)	-
EV characterization	No characterization	Nano particle tracking or dynamic light scatter	Nano particle tracking and western blot OR microscopy	Electron microscopy and particle tracking	At least 1 other methods, plus other novel methods (inc. FACS)	-
Sample type	-	2D cell line	3D cell lines	Animal models	Human samples	Human samples and databases of human samples

**Table 2:** Inclusion and Exclusion criteria used for study selection.

Inclusion Criteria	Exclusion Criteria
Research involving the EV contents, EV effects on recipients or microenvironment AND/OR therapeutically targeting EV derived mechanism of CSCs	Non-original research paper (reviews, commentary, case report)
Included isolation/characterization methods for EV collection	Articles published in a language other than English
	No specific EV extraction method or protocol
	Methodology Focus

## Data Availability

Data sharing was not applicable. The data presented in this study are available as published studies. No new data was presented.

## Supplementary Materials

The following are available online at https://www.mdpi.com/article/10.3390/cancers13102435/s1, Table S1: Assessment of studies reporting the content of EVs and their effects. Table S2: Assessment of studies reporting pathways activated by CSC EVs. Table S3: Assessment of studies reporting CSC EV directed therapeutics.
