# Supplementary material for: The Role of Extracellular Vesicles in the Development of a Cancer Stem Cell Microenvironment Niche and Potential Therapeutic Targets: A Systematic Review
Source: Cancers (Basel). 2021 May 18;13(10):2435. doi: 10.3390/cancers13102435 (PMC8157362; doi:10.3390/cancers13102435)
Supplement: Supplementary file 1 [file cancers-13-02435-s001.zip › cancers-1164604-supplementary-final-final.pdf]

The Role of Extracellular Vesicles in the Development of a Cancer Stem Cell Microenvironment Niche and Potential Therapeutic Targets: A Systematic Review

Thomas J Brown and Victoria James

Table S1. Summary of studies on the content of EVs in cancer stem cells.

Extracellular Vesicle Cargo						
**Author, Year**	**Title**	**Aim**	**Cancer**	**Biological focus**	**Samples**	**Conclusion**
Hardin *et al.*, 2018 [1]	Thyroid cancer stem-like cell exosomes: regulation of EMT via transfer of lncRNAs	How exosomes lncRNAs facilitate the cancer cell stemness and the EMT process in the aggressive anaplastic thyroid carcinoma	Anaplastic thyroid carcinoma	lncRNAs	*In vitro* 3D Cell Culture	Exosomes to communicate with and modulate adjacent and distant tumour microenvironment through the transfer of ncRNAs. Specifically, in the transfer of lncRNA, linc-ROR, induceing CSC or EMT
Donnarumma *et al.*, 2017 [2]	Cancer-associated fibroblasts release exosomal microRNAs that dictate an aggressive phenotype in breast cancer	To view the miRNA mediators within Cancer-associated fibroblasts exosomes that promote breast cancer stem cellness	Breast cancer	miRNAs	*In vitro* 3D Cell culture *In vivo* Human	miRs -21, -378e, and -143, found in exosomes promoted the stemness and EMT phenotype of breast cancer cells and CAFs regulate the development of an aggressive phenotype in breast cancer cells through exosome-mediated delivery of oncogenic miRs.
Razmkhah *et al.*, 2017 [3]	Leukaemia microvesicles affect healthy hematopoietic stem cells	Investigate the effect of leukaemia microvesicles on healthy umbilical cord blood hematopoietic stem cells to find evidence of cell information transferring	Leukaemia	miRNAs	*In vitro* 2D Cell culture *In vivo* Human	Leukaemia microvesicles are able to induce some effects on healthy hematopoietic stem cells such as promoting cell survival and some microRNAs deregulation, while stemness is maintained. miRNA 21 +29a

Ramteke *et al.*, 2013 [4]	Exosomes secreted under hypoxia enhance invasiveness and stemness of prostate cancer cells by targeting adherens junction molecules	Investigating the role of exosomes from hypoxic PCA cells in enhancing the invasiveness and stemness of naïve PCA cells, as well as in promoting cancer-associated fibroblast phenotype in prostate stromal cells	Prostate cancer	Protein	*In vitro* 3D cell culture	Hypoxic exosomes are loaded with unique proteins that could enhance invasiveness, stemness, and induce microenvironment changes

Table S2. Summary of studies on the pathways activated by CSC EV mediated communication.

**Activated Pathways**						
**Author, Year**	**Title**	**Aim**	**Cancer**	**Pathway**	**Samples**	**Conclusion**
Hwang *et al.*, 2019 [5]	Tumor stem-like cell-derived exosomal RNAs prime neutrophils for facilitating tumorigenesis of colon cancer	the mechanism governing colorectal cancer CSC-regulated neutrophil expansion	Colorectal cancer	NF-κB	*In vitro* 3D Cell culture *In vivo* Human Mouse	Describes the heterogeneity of tumor exosomes and elucidates a unique behavior of tumor exosomal RNAs in the establishment of a pro-tumoral microenvironment through signaling axis.
Gu *et al.*, 2016 [6]	Exosomes derived from human mesenchymal stem cells promote gastric cancer cell growth and migration via the activation of the Akt pathway	To investigate the effects of MSC-ex on the malignant/stemness properties of gastric cancer cells	Gastric Cancer	protein kinase B/ak-t signalling pathway	*In vitro* 2D Cell culture	MSC-ex promoted the proliferative and metastatic potential of gastric cancer cells ex vivo via the induction of the epithelial-mesenchymal transition and stemness via the protein kinase B/akt signaling pathway
Liu *et al.*, 2020 [7]	Cancer associated fibroblasts-derived exosomes contribute to radioresistance through promoting colorectal cancer stem cells phenotype	Investigate whether exosomes derived from CAFs (CAF-exosomes) are involved in mediating resistance to radiotherapy in colorectal cancer and to explore the underlying mechanism.	Colorectal cancer	TGF-β signalling pathway	*In vitro* 2D Cell culture *In vivo* Mouse	CAFs promote stemness of CRC cells and thus increase radiation resistance. Exosomes derived from CAFs play a crucial role through activating TGF-β signaling pathway in this process.
Ren *et al.*, 2018 [8]	Carcinoma-associated fibroblasts promote the stemness and chemoresistance of colorectal cancer by transferring exosomal lncRNA H19.	To investigate the molecular mediators within carcinoma associated fibroblasts exosomes, and how these promote stem cellness and chemoresistance	Colorectal cancer	β-catenin pathway	*In vitro* 2D Cell culture *In vivo* Mouse	CAFs promote the stemness and chemoresistance of CRC by transferring exosomal H19. H19 activated the β-catenin pathway via acting as a competing endogenous RNA sponge for miR-141, while miR-141 inhibited the stemness of CRC cells. H19 expressed by CAFs of the colorectal tumor stroma contributes

						to tumor development and chemoresistance
Cheng *et al.*, 2019 [9]	RAB27B-activated secretion of stem-like tumor exosomes delivers the biomarker microRNA-146a-5p, which promotes tumorigenesis and associates with an immunosuppressive tumor microenvironment in colorectal cancer	Elucidating miRNA mechanisms of tumor exosome-mediated stemness expansion	Colorectal cancer	B-catenin/Tcf-4-activated RAB27B expression	*In vitro* 3D Cell culture *In vivo* Human	miRNA-146a-5p (miR-146a) in CRCSC exosomes promotes stem-like properties and tumorigenicity by targeting Numb in recipient CRC cells. Leading to increased neutrophils and decreased t cells in Colorectal cancer patients
Mao *et al.*, 2017 [10]	UBR2 Enriched in p53 Deficient Mouse Bone Marrow Mesenchymal Stem Cell-Exosome Promoted Gastric Cancer Progression via Wnt/β-Catenin Pathway	Investigate the regulation of ubiquitin protein ligase E3 component n-recognin 2 (UBR2) enriched in exosomes secreted by p53 deficient mouse bone marrow MSC (p53-/- mBMMSC) in gastric cancer progression in vivo and in vitro	Gastric cancer	Wnt/β-catenin pathway	*In vitro* 2D Cell culture *In vivo* Mouse	p53-/- mBMMSC exosomes could deliver UBR2 to target cells and promote gastric cancer growth and metastasis by regulating Wnt/β-catenin pathway
Li and li 2018 [11]	Exosomes from BM-MSCs increase the population of CSCs via transfer of miR-142-3p	To elucidate how BM-MSCs influence the stemness of recipient colon cancer cells and how this is facilitated by exosome miRNA contents	Colon cancer	Numb/ Notch signalling pathway	*In vitro* 2D Cell culture *In vivo* Mouse Human	Findings indicate that BM-MSC-derived exosomes promote colon cancer stem cell-like traits via increased miR-142-3p via the numb/notch signalling pathway
Sun *et al.*, 2020 [12]	Glioblastoma Stem Cell-Derived Exosomes Enhance Stemness and Tumorigenicity of Glioma Cells by Transferring Notch1 Protein	Investigate whether GSC exosomes could reprogramme non-GSC glioma cells into GSCs, and to explore its potential mechanism involved.	Glioblastoma	Notch 1 signalling	*In vitro* 3D Cell culture	GSC exosomes act as information carriers, mediated non-GSC glioma cell dedifferentiation into GSCs by delivering Notch1 protein through Notch1 signaling activation, and enhanced stemness and tumorigenicity of non-GSC glioma cells.

Table S3. Summary of the studies in CSC EV directed therapeutics.

CSC Targeted Therapeutics						
**Author, Year**	**Title**	**Aim**	**Cancer**	**Therapeutic**	**Samples**	**Conclusion**
Chen *et al.*, 2020 [13]	Ovatodiolide Suppresses Oral Cancer Malignancy by Down-Regulating Exosomal Mir-21/STAT3/β-Catenin Cargo and Preventing Oncogenic Trans-	Explored the role of cancer stem cell-derived extracellular vesicles (CSC_EVs) generated from CAL27 and SCC-15 OSCC cells in the devel-	Oral squamous cell carcinoma	Ovatodiolide	*In vitro* 3D Cell Culture Primary Cell Culture *Additional* Database	Findings show Preclinical evidence that OV treatment suppresses tumorigenesis and OSCC stemness, as well as normalizes the TME, by

	formation of Normal Gingival Fibroblasts	opment of cisplatin (CDDP) resistance.				reducing the oncogenic cargo in CSC-EVs
Gernapudi *et al.*, 2015 [14]	Targeting exosomes from preadipocytes inhibits preadipocyte to cancer stem cell signaling in early-stage breast cancer	investigated the role of exosomes secreted from preadipocytes in regulating cancer cell behavior and tumor formation	Breast cancer	Shikonin	*In vitro* 2D Cell Culture 3D Cell Culture *In vivo* Mouse Models	miR-140/ SOX2/SOX9 axis regulates differentiation, stemness, and migration in the tumor microenvironment. preadipocyte-derived exosomes promote tumorigenesis in vivo, with Treatment with shikonin inhibiting preadipocyte signaling.
Xing *et al.*, 2018 [15]	Loss of XIST in breast cancer activates MSN-c-Met and reprograms microglia via exosomal microRNA to promote brain metastasis	Exploring the potential role and mechanisms by which lncRNAs found in EVs act in promoting the metastasis of breast cancer to the brain through cancer stem cellness	Breast cancer	Fludarabine	*In vitro* 2D Cell Culture *In vivo* Mouse Models Human *Additional* Human databases	The loss of the lncRNA XIST promotes brain metastasis in breast cancer and indentifies as a potential therapeutic agent that specifically eliminates XISTlow tumor cells in the breast and brain.
Chuang *et al.*, 2020. [16]	Preclinical Evidence of STAT3 Inhibitor Pacritinib Overcoming Temozolomide Resistance via Downregulating miR-21-Enriched Exosomes from M2 Glioblastoma-Associated Macrophages	To explore the role of signaling molecules such as cytokines, proteins, and microRNAs in promotion of stemness features and the ability of Pacritinib in targeting the CSC alterations	Glioblastoma multiforme	Pacritinib	*In vitro* 3D Cell Culture *In vivo* Mouse Human	Findings show potential of pacritinib alone or in combination with TMZ to suppress GBM tumorigenesis via modulating STAT3/miR-21/PDCD4 signaling.

References

1. Wang, J.; Li, D.; Cang, H.; Guo, B. Crosstalk between cancer and immune cells: Role of tumor-associated macrophages in the tumor microenvironment. *Cancer Med.* **2019**, *8*, 4709–4721.
2. Wieckowski, E.U.; Visus, C.; Szajnik, M.; Szczepanski, M.J.; Storkus, W.J.; Whiteside, T.L. Tumor-derived microvesicles promote regulatory T cell expansion and induce apoptosis in tumor-reactive activated CD8+ T lymphocytes. *J. Immunol.* **2009**, *183*, 3720–3730.
3. Hardin, H.; Helein, H.; Meyer, K.; Robertson, S.; Zhang, R.; Zhong, W.; Lloyd, R.V. Thyroid cancer stem-like cell exosomes: Regulation of EMT via transfer of lncRNAs. *Lab. Investig.* **2018**, *98*, 1133–1142.
4. Han, L.; Lam, E.W.-F.; Sun, Y. Extracellular vesicles in the tumor microenvironment: Old stories, but new tales. *Mol. Cancer* **2019**, *18*, 59.

5. Cheng, W.C.; Liao, T.T.; Lin, C.C.; Yuan, L.T.E.; Lan, H.Y.; Lin, H.H.; Teng, H.W.; Chang, H.C.; Lin, C.H.; Yang, C.Y. RAB27B-activated secretion of stem-like tumor exosomes delivers the biomarker microRNA-146a-5p, which promotes tumorigenesis and associates with an immunosuppressive tumor microenvironment in colorectal cancer. *Int. J. Cancer* **2019**, *145*, 2209–2224.
6. Razmkhah, F.; Soleimani, M.; Mehrabani, D.; Karimi, M.H.; Amini Kafi-abad, S.; Ramzi, M.; Iravani Saadi, M.; Kakoui, J. Leukemia microvesicles affect healthy hematopoietic stem cells. *Tumor Biol.* **2017**, *39*, 1010428317692234.
7. Hwang, W.-L.; Lan, H.-Y.; Cheng, W.-C.; Huang, S.-C.; Yang, M.-H. Tumor stem-like cell-derived exosomal RNAs prime neutrophils for facilitating tumorigenesis of colon cancer. *J. Hematol. Oncol.* **2019**, *12*, 1–17.
8. Liu, L.; Zhang, Z.; Zhou, L.; Hu, L.; Yin, C.; Qing, D.; Huang, S.; Qin, J.; Cai, X.; Chen, Y. Cancer associated fibroblasts-derived exosomes contribute to radioresistance through promoting colorectal cancer stem cells phenotype. *Exp. Cell Res.* **2020**, 111956.
9. Ramteke, A.; Ting, H.; Agarwal, C.; Mateen, S.; Somasagara, R.; Hussain, A.; Graner, M.; Frederick, B.; Agarwal, R.; Deep, G. Exosomes secreted under hypoxia enhance invasiveness and stemness of prostate cancer cells by targeting adherens junction molecules. *Mol. Carcinog.* **2015**, *54*, 554–565.
10. Li, H.; Li, F. Exosomes from BM-MSCs increase the population of CSCs via transfer of miR-142-3p. *Br. J. Cancer* **2018**, *119*, 744–755.
11. Gu, H.; Ji, R.; Zhang, X.; Wang, M.; Zhu, W.; Qian, H.; Chen, Y.; Jiang, P.; Xu, W. Exosomes derived from human mesenchymal stem cells promote gastric cancer cell growth and migration via the activation of the Akt pathway. *Mol. Med. Rep.* **2016**, *14*, 3452–3458.
12. Mao, J.; Liang, Z.; Zhang, B.; Yang, H.; Li, X.; Fu, H.; Zhang, X.; Yan, Y.; Xu, W.; Qian, H. UBR2 Enriched in p53 Deficient Mouse Bone Marrow Mesenchymal Stem Cell-Exosome Promoted Gastric Cancer Progression via Wnt/β-Catenin Pathway. *Stem Cells* **2017**, *35*, 2267–2279.
13. Ren, J.; Ding, L.; Zhang, D.; Shi, G.; Xu, Q.; Shen, S.; Wang, Y.; Wang, T.; Hou, Y. Carcinoma-associated fibroblasts promote the stemness and chemoresistance of colorectal cancer by transferring exosomal lncRNA H19. *Theranostics* **2018**, *8*, 3932.
14. Chen, J.-H.; Wu, A.T.; Bamodu, O.A.; Yadav, V.K.; Chao, T.-Y.; Tzeng, Y.-M.; Mukhopadhyay, D.; Hsiao, M.; Lee, J.-C. Ovatodiolide suppresses oral cancer malignancy by down-regulating exosomal mir-21/STAT3/β-catenin cargo and preventing oncogenic transformation of normal gingival fibroblasts. *Cancers* **2020**, *12*, 56.
15. Chuang, H.-Y.; Su, Y.-k.; Liu, H.-W.; Chen, C.-H.; Chiu, S.-C.; Cho, D.-Y.; Lin, S.-Z.; Chen, Y.-S.; Lin, C.-M. Preclinical evidence of STAT3 inhibitor pacritinib overcoming temozolomide resistance via downregulating miR-21-enriched exosomes from M2 glioblastoma-associated macrophages. *J. Clin. Med.* **2019**, *8*, 959.
16. Sun, Z.; Wang, L.; Zhou, Y.; Dong, L.; Ma, W.; Lv, L.; Zhang, J.; Wang, X. Glioblastoma Stem Cell-Derived Exosomes Enhance Stemness and Tumorigenicity of Glioma Cells by Transferring Notch1 Protein. *Cell. Mol. Neurobiol.* **2019**, *40*, 767–784.
